# Eliminating the Effect of Image Border with Image Periodic Decomposition for Phase Correlation Based Remote Sensing Image Registration †

**DOI:** 10.3390/s19102329

**Published:** 2019-05-20

**Authors:** Yunyun Dong, Weili Jiao, Tengfei Long, Lanfa Liu, Guojin He

**Affiliations:** 1 Aerospace Information Research Institute, Chinese Academy of Sciences, Beijing 100094, China; dongyunyun14@mails.ucas.ac.cn (Y.D.); hegj@radi.ac.cn (G.H.); 2 University of Chinese Academy of Sciences, Beijing 100049, China; 3 Hainan Key Laboratory for Earth Observation, Sanya 572029, China; 4 Institute for Cartography, TU Dresden, 01062 Dresden, Germany; lanfa.liu@mailbox.tu-dresden.de

**Keywords:** image registration, Phase-correlation, Image peiodic decomposition

## Abstract

In the remote sensing community, accurate image registration is the prerequisite of the subsequent application of remote sensing images. Phase correlation based image registration has drawn extensive attention due to its high accuracy and high efficiency. However, when the Discrete Fourier Transform (DFT) of an image is computed, the image is implicitly assumed to be periodic. In practical application, it is impossible to meet the periodic condition that opposite borders of an image are alike, and image always shows strong discontinuities across the frame border. The discontinuities cause a severe artifact in the Fourier Transform, namely the known cross structure composed of high energy coefficients along the axes. Here, this phenomenon was referred to as effect of image border. Even worse, the effect of image border corrupted its registration accuracy and success rate. Currently, the main solution is blurring out the border of the image by weighting window function on the reference and sensed image. However, the approach also inevitably filters out non-border information of an image. The existing understanding is that the design of window function should filter as little information as possible, which can improve the registration success rate and accuracy of methods based on phase correlation. In this paper, another approach of eliminating the effect of image border is proposed, namely decomposing the image into two images: one being the periodic image and the other the smooth image. Replacing the original image by the periodic one does not suffer from the effect on the image border when applying Fourier Transform. The smooth image is analogous to an error image, which has little information except at the border. Extensive experiments were carried out and showed that the novel algorithm of eliminating the image border can improve the success rate and accuracy of phase correlation based image registration in some certain cases. Additionally, we obtained a new understanding of the role of window function in eliminating the effect of image border, which is helpful for researchers to select the optimal method of eliminating the effect of image border to improve the registration success rate and accuracy.

## 1. Introduction

Image registration is a core technology of image processing, particularly widely applied in the remote sensing community, such as change detection [1,2], image fusion, long-time remote sensing data analysis, surface displacement of landslides [3,4], photogrammetry [5], etc. According to the comprehensive overview of image matching approaches [6], image registration methods can be grouped into two categories: feature based registration methods and area based registration methods. For the feature based image registration methods, according to the utilized features, single pixel feature and multipixel feature, they can loosely be grouped into two categories. For the first category, its feature is the single pixel feature, such as ground control point (GCP). Its main procedures include feature detection, feature matching, transform model estimation and image warping [7,8,9,10]. Among these procedures, feature detection and feature matching are two core steps, and they are also two steps to which researchers devote much effort [11]. After decades of development, feature detection approaches have developed from the initial handcrafted methods (Difference of Gaussian (DoG) [12], Harris [13], Hessian [14] and Wavelet Features.) to the latest deep learning based approaches (TILDE [15], CovDet [16] and SuperPoint [17]). For feature matching, the feature descriptors are vital for correct correspondence. Feature descriptors are also developed from hand-engineered descriptors (HoG [18], DAISY [19], etc.) to deep learning based descriptors (DDesc [20], L2-Net [21] and HardNet [22]). Although the deep learning based feature detectors and descriptors are promising, the lack of available public remote sensing image matching data makes it hard to go beyond the hand-engineered approaches for the time being. Thus, the handcrafted features and descriptors are also the mainstream methods. However, these methods are originated from the field of computer vision and they have encountered many challenging problems when applied in the community of remote sensing images. Specially, the nonlinear radiation distortions of remote sensing images result in a dramatic drop in performance, even failure.

For the second category, its feature is multipixel features, such as lines and polygons. Here, we mainly introduce the multipixel feature captured by the Wavelet and Shearlet transform. Its main procedures include the selection of feature space, extraction of features, the selection of similarity metric and search strategy [23,24,25,26]. For the features identified by wavelet transform, they are isotropic textural features. For the features identified by Shearlet transform, they are anisotropic features, such as edges, roads, rivers and other edge-like features. The combination of features captured by the wavelet transform and Shearlet transform is more robust to the initial choice of global transform [26]. In addition, the multiresolution nature of wavelet decomposition can accelerate the calculation of geometric transform model. In fact, these multipixel features are matched with an optimization scheme. Due to the non-convex characteristic of objective function, there exist some limitations of feature space in the practical application. For example, it is very difficult to conduct in the search space that consists of polynomial transformations to address more complex and spatially varying distortions within images. In addition, the final registration result is also sensitive to the similarity measurement and optimization method.

In recent years, image registration based on phase correlation has drawn extensive attention due to its three merits: high registration accuracy and robustness to noise and variation of gray value, constant processing time for the fixed size image, and ease of implementation and parallelization. The existing methods based on phase correlation can loosely be grouped into two categories. One category is determining the translation by precisely locating the main peak of Inverse Fourier Transform of the normalized cross-power. The other category is directly estimating the linear phase difference in the frequency domain. For the first category, the commonly used solutions are interpolation methods, such as 1D parabolic function, sinc function, modified sinc function and Gaussian function [27,28,29]. However, the shortcomings of the method are sensitivity to noise and other errors. For the other category, the principle behind it is that the phase difference in the frequency domain is linear with respect to displacement in the spatial domain. The linear phase difference can be achieved by different approaches, such as least square fitting [30], the combination of singular value decomposition and random sample consensus algorithm [31], and low-rank factorization by modeling the noise with Gaussian mixtures model [32]. The sub-pixel accuracy of phase correlation-based image registration is mainly affected by the noise, aliasing and border of the image. As suggested in [30], abandoning Inverse Fourier Transform can alleviate the side effect caused by this noise and aliasing. Thus, most researchers have focused on precisely estimating the linear phase difference in the frequency domain rather than identifying the location of main peak by the introduction of Inverse Fourier Transform.

As for the effect of image border, it is caused by the implicit assumption of periodicity in the computation of Discrete Fourier Transform of an image. In almost all circumstances, it is not reasonable to assume that the opposite border of image is similar, so the assumed periodic image will lead to strong discontinuities across the image border. For the effect produced by the border of image, the commonly used approach is weighting the reference and sensed images with a window function in the spatial domain separately. However, the operation of weighting window function can cause additional negative effects, namely reducing the common and useful information for registration based on phase correlation, especially in the case of small patch-based matching or small overlap existence between image pairs.

To deal with the effect of image border, we applied a novel approach, image periodic decomposition method (decomposition method for short) to decompose the original image into two components, namely periodic image component and smooth image component. Then, we replaced the original image by the periodic image component, which can eliminate the effect of image border effectively and there is almost no loss of image information at the same time. Thus, the decomposition method can improve the estimation accuracy and success rate of image registration based on phase correlation, especially in the case of small image patches’ registration.

In this paper, our main contributions are two-fold: (1) a novel approach, image decomposition was utilized to eliminate the effect of image border, which degrades the accuracy and success rate of phase correlation-based image registration. Compared to the traditional weighted filtering approaches, the image decomposition method can not only eliminate the effect of image border, but also it rarely reduces information away from the image border. Thus, the decomposition method can improve the registration accuracy and success rate in some certain cases. Furthermore, it is easy to use and there are no parameters to tune. (2) We introduced a new concept of SSNR (Similar-to-Signal-Noise Ratio) to obtain a new view of the role of weighting window function; it can be more helpful to guide researchers to select a proper approach for eliminating the effect of image border to improve the registration accuracy and success rate in the practical applications.

This paper extends a preliminary version of [33] by adding: (1) a detailed description of various methods of eliminating the effect of image border and the phenomenon of the effect of image border; (2) two groups of experiments about displacement estimation success rate and accuracy to further illustrate the advantages of the proposed method, and a further analysis of elimination of the effect of image border methods; (3) a thorough discussion of elimination of the effect of image border, and a new understanding of the role of window function.

The remainder of this paper is organized as follows. Section 2 introduces the related work of elimination of the effect of image border. Section 3 details the principle of image registration based on phase correlation and our proposed method of decomposition method. In Section 4, three groups of experiments were carried out to illustrate the advantages of the decomposition method, compared to three other window functions, and further analysis was made to give more insights on eliminating the effect of image border. The discussion is given in Section 5. The conclusions are drawn in Section 6.

## 2. Related Work

For the implicit periodicity property of Discrete Fourier Transform (DFT) and the nature of the image for which the opposite borders are not alike, the effect of image border always occurs in the process of image registration based on phase correlation. In the past research, various approaches are proposed to deal with the problem. Among them, three approaches are popular, namely Blackman window, raised-cosine window [3] and flap-top window [34].

For the Blackman window of length *N*, its formula in one dimension is defined as follows:

(1) $$w\left(n\right)=0.42-0.5\cos\frac{2\pi n}{N-1}+0.08\cos\frac{4\pi n}{N-1},0\leq n\leq N-1.$$

**Figure 1:**
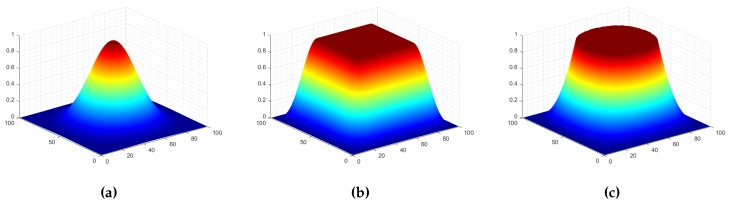
The three-dimensional diagrams of different window functions. (**a**) is the diagram of Blackman window function; (**b**) is the diagram of raised-cosine window function; (**c**) is the diagram of flap-top window function. The length of all window functions is 100. For raised-cosine window function, its roll-off factor β is set to 0.25. For the flap-top window function, its stretch factor *k* is set to 2.7.

It is a commonly used window function when using spectral analysis in the signal processing field. Its three-dimensional diagram is shown in Figure 1a. Apparently, for an image patch weighted by the Blackman window, the discontinuities across the image border are smoothed out, but a significant amount of signal is also degraded.

Another window function, named raised-cosine [3], was proposed to achieve a good balance between reducing effect of image border and the information retention of image. The one-dimensional expression of the raised-cosine window of length *N* is given as follows:

(2) $$w\left(n_{1},n_{2}\right)=\left\{\begin{array}{cc} 1, & \Gamma (n_{1},n_{2})\geq 1,\\ \Gamma (n_{1},n_{2}), & otherwise, \end{array}\right.$$

where $\Gamma (n_{1},n_{2})=k\cdot 0.5\left(1-\cos\left(2\pi \left(n_{1}/M\right)\right)\right)\cdot 0.5\left(1-\cos\left(2\pi \left(n_{2}/N\right)\right)\right)$, *M*, *N* are the sizes of the images, *k* is the stretch factor. A three-dimensional diagram of it is shown in Figure 1b.

**Figure 2:**
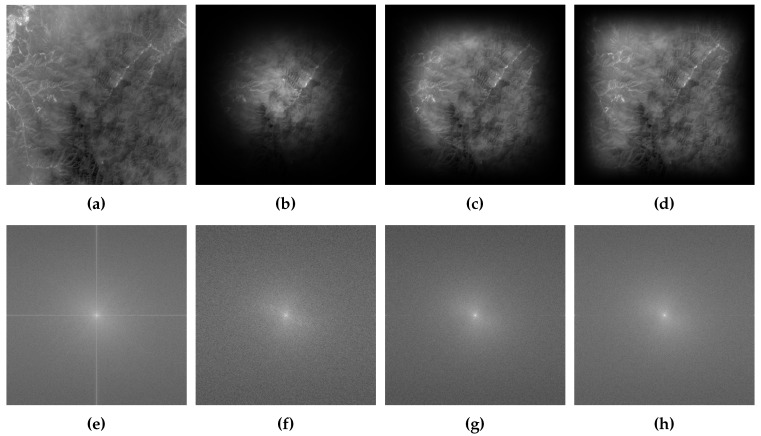
The resulting images of image filtered by different window functions and the corresponding amplitude spectrum. (**a**) is the original image, its size is 1000×1000 pixels. (**b**–**d**) are the result images after filtering by Blackman, flap-top and raised-cosine window function, respectively. (**e**–**h**) are the corresponding amplitude spectrum of (**a**–**d**), respectively. It is worth noting that the amplitude spectrum was operated logarithmically for clear presentation.

The role of the aforementioned weighting window function is similar to the general filter in the spatial domain. The essences of these window functions are progressively making the border of the image fuzzy and preserving the content away from the border of image. In fact, in order to avoid adding new discontinuous information to the images, some content near the border will be subjected to varying degrees of fuzziness. The difference between the three window functions is how much the information near the image border is processed. It was further illustrated by the result of an image filtered by different window functions, as shown in Figure 2. Here, the parameters of flat-top and raised-cosine window function are the same as ones in the Figure 1. Figure 2a is the original image, Figure 2b–d are the resulting images after filtering by Blackman, flat-top and raised-cosine window function, respectively. Figure 2e–h are the amplitude spectrum of Figure 2a–d, respectively. From the bottom row in Figure 2, the cross structure is distinct in the amplitude spectrum of original image, and disappeared in other three amplitude spectrums. This shows that the weighting window function can eliminate the effect of image border. However, for the Blackman window, it blurs out a large amount of the content of images and retains a fraction of content centered at the image unchanged. Blurring out a large amount of content means that some valuable information is degraded for registration based on phase correlation. This will reduce the accuracy of registration due to little common information left. For raised-cosine and flap-top, they blur the borders while keeping the content in the middle of the image unchanged as much as possible. However, some contents near the border are also degraded inevitably. Especially when the overlap of the image pairs is small, there is a dilemma that the common areas of image pairs are fuzzy and the common information is reduced to only a little. Consequently, this will lead to the failure of registration based on phase correlation due to the lack of a large amount of the common information.

Here, we aimed to solve the problem of effect of image border. A corresponding strategy is decomposition of an image. The concrete decomposition approach is decomposing the image into two images: one is periodic image and the other is smooth image. We finally discarded the smooth image and calculated the Discrete Fourier Transform only for the periodic image. This method only eliminates the effect of image border and rarely degrades the content near the image border.

## 3. Methodology

In this section, firstly, a principle of phase correlation based image registration is introduced. Secondly, the details of eliminating the effect of image border and the image decomposition method were given. The periodic decomposition of an image can be reformulated as a unique solver for a specified problem [35]. The decomposition operation is performed by using the classic Fourier Transform based Poison solver. Thirdly, a framework of phase correlation based image registration was outlined.

### 3.1. The Principle of Image Registration Based on Phase Correlation

The basic theory of image registration based on phase correlation is Fourier shift theorem. Suppose that $f_{1}\left(x,y\right)$ and $f_{2}\left(x,y\right)$ denote the reference image and sensed image, respectively. In addition, they satisfy the following relationship:

(3) $$f_{1}\left(x,y\right)=f_{2}\left(x-x_{0},y-y_{0}\right),$$

where $x_{0}$, $y_{0}$ is the displacement in the *x*- and *y*-directions, respectively. According to the Fourier shift theorem [1], the normalized cross-power spectrum matrix can be expressed as follows:

(4) $$\begin{array}{cc} Q(\xi_{x},\xi_{y}) & =\frac{F_{2}\left(\xi_{x},\xi_{y}\right)F_{1}{\left(\xi_{x},\xi_{y}\right)}^{*}}{|F_{1}\left(\xi_{x},\xi_{y}\right)F_{2}\left(\xi_{x},\xi_{y}\right)|}\\ & =\exp\left(-i\left(\xi_{x}x_{0}+\xi_{y}y_{0}\right)\right)\\ & =\exp\left(-i\left(\xi_{x}x_{0}\right)\right)\exp\left(-i\left(\xi_{y}y_{0}\right)\right)\\ & =q_{x_{0}}\left(\xi_{x}\right)q_{y_{0}}\left(\xi_{y}\right), \end{array}$$

where $\xi_{x}$ and $\xi_{y}$ denote the frequency component, and ∗ denotes the complex conjugate operation. The magnitude of $Q(\xi_{x},\xi_{y})$ is normalized to 1 for any frequency component. It is therefore insensitive to radiation variation and image content, which makes the phase correlation based image registration method more suitable to matching multi-sensors’ images. Apparently, the phase difference is linear with respect to each frequency component, and the displacement in the *x*- and *y*-directions can be expressed with the slope of the unwrapped phase angles of components $q_{x_{0}}\left(\xi_{x}\right)$ and $q_{y_{0}}\left(\xi_{y}\right)$, respectively. Then, the displacement $x_{0}$, $y_{0}$ can be written as follows:

(5) $$x_{0}=\frac{s_{x}M}{2\pi },y_{0}=\frac{s_{y}N}{2\pi },$$

where *M* and *N* denote the size of the image.

When the image pair has the similarity transformation relationship, they have the following relationship in the spatial domain:

(6) $$\begin{array}{c} f_{1}\left(x,y\right)=f_{2}(s\left(x\cos\theta_{0}+y\sin\theta_{0}\right)+\Delta x,\\ s(-x\sin\theta_{0}+y\cos\theta_{0})+\Delta y), \end{array}$$

where $\theta_{0}$ represents the rotation angle, *s* represents the scalar, Δ*x* and Δ*y* represent the displacement in the *x*- and *y*-directions, respectively. After applying Fourier Transform to the image pair, we have the following equation:

(7) $$\begin{array}{c} F_{1}\left(\xi_{x},\xi_{y}\right)=s^{-2}KF_{2}(s^{-2}\left(\xi_{x}\cos\theta_{0}+\xi_{y}\sin\theta_{0}\right),\\ s^{-2}\left(-\xi_{x}\sin\theta_{0}+\xi_{y}\cos\theta_{0}\right)), \end{array}$$

where $\xi_{x}$, $\xi_{y}$ denote the frequency components, $K=e^{-i(\Delta x\left(\xi_{x}\cos\theta_{0}+\xi_{y}\sin\theta_{0}\right)+\Delta y\left(-\xi_{x}\sin\theta_{x}+\xi_{y}\cos\theta_{0}\right))}$. According to the relationship between Cartesian and Polar coordinates, $\xi_{x}=r\cos\theta$, $\xi_{y}=r\sin\theta$, and, calculating the magnitudes $M_{1}$ and $M_{2}$ of each side of the equation (7), respectively, a relationship of amplitude of $F_{1}$ and $F_{2}$ was derived as follows:

(8) $$M_{1}{\left(r,\theta \right)}^{-2}=M_{2}\left(s^{-1}r,\theta +\theta_{0}\right).$$

Furthermore, apply logarithm transform to the radius *r*, and the final equation is acquired as follows:

(9) $$M_{1}\left(logr,\theta \right)=M_{2}\left(logr-logs,\theta +\theta_{0}\right).$$

Obviously, the rotation angle θ and scalar *s* can be acquired by applying the phase correlation to $M_{1}$ and $M_{2}$ again.

In short, for only displacement estimation, the phase correlation based method can estimate the displacement directly. For angle and scale estimation, a log-polar Fourier transform is first necessary to operate on the reference and sensed image, respectively. Then, the scale and angle can be converted to the displacements between the amplitude spectrum of log-polar Fourier transform of reference and sensed images [36].

### 3.2. Eliminating the Effect of Image Border

**Figure 3:**
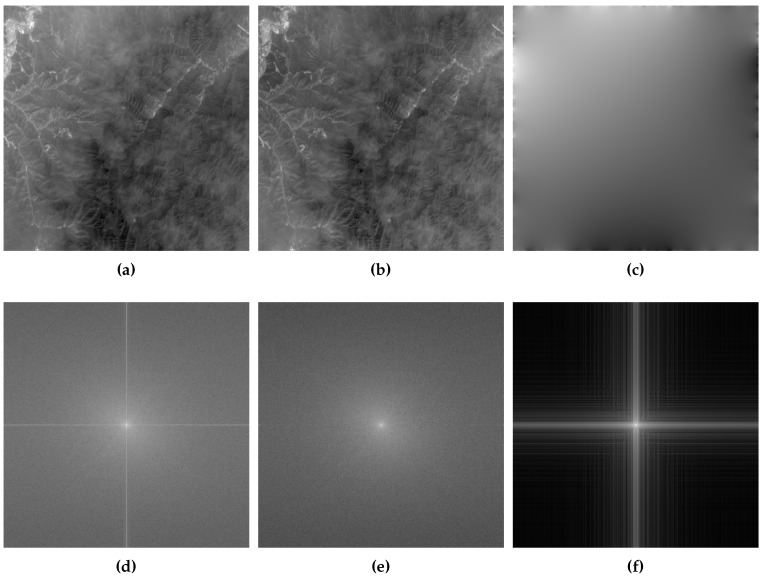
Image decomposition and its corresponding amplitude spectrum of image. (**a**–**c**) are original image, periodic image and smooth image, respectively. (**d**–**f**) are the corresponding amplitude spectrum of (**a**–**c**), respectively. Compared to (**d**) of the amplitude spectrum of original image, the cross structure in the (**a**) of the amplitude spectrum of the periodic image disappears visually. It is noteworthy that, for other images, the cross structure may not vanish completely because the cross structure contains two pieces of information: one is produced by the discontinuity of image border, and the other is the content of the image itself.

The principle behind the effect of the image border is the implicit assumption of periodicity in the calculation of Discrete Fourier Transform and the extrinsic representation is the well-known cross structure in the magnitude of the frequency spectrum, as shown in Figure 3d. The high energy coefficients along the axes will reduce the accuracy of image registration heavily. Here, to eliminate the effect of image border, we will decompose the original image into two images: one is the periodic image, which is closest to the original image [35], the other is the smooth image, whose gray value varies slowly except for the near area of image border. In the practical application, we apply Discrete Fourier Transform only to the periodic image, which can effectively eliminate the effect of image border. The basic principle of the image decomposition is the periodic component of an image; *p* is the unique solution of the following problem [35]:

(10) $$\left\{\begin{array}{cc} \Delta p & {=\Delta }_{i}I,\\ mean(p) & =mean(I), \end{array}\right.$$

where Δ denotes the usual discrete periodic Laplacian (each point has four neighbors) and $\Delta_{i}$ denotes the discrete Laplacian in the interior of the image domain (points at the border of the image have only two or three neighbors). The solution *p* is acquired by the classical Fast Fourier Transform-based Poisson solver, which consists of four steps:

(1) Calculate the Discrete Laplacian $\Delta_{i}I$ of original image I;
(2) Calculate the Discrete Fourier Transform $\tilde{\Delta I}$ of $\Delta_{i}I$;
(3) Calculate the Discrete Fourier Transform $\tilde{p}$ of p by the usage of inversing Discrete periodic Laplacian:
  (11) $$\left\{\begin{array}{cc} \tilde{p}\left(\xi_{x},\xi_{y}\right) & =\frac{1}{4-2\cos\left(\frac{2\xi_{x}\pi }{M}\right)-2\cos\left(\frac{2\xi_{2}\pi }{N}\right)}\tilde{\Delta I}\left(\xi_{1},\xi_{2}\right),\\ \tilde{p}\left(0,0\right) & =\sum_{x,y}I\left(x,y\right), \end{array}\right.$$
(4) Apply inverse Discrete Fourier Transform to $p(\xi_{1},\xi_{2})$ and acquire the periodic image *p*.

After decomposing the reference and sensed images into two images, the reference and sensed images are replaced with its corresponding periodic images. As shown in Figure 3, the periodic image (b) is visually close to its corresponding original image, and the gray value of smooth image (c) varies smoothly except for the area around the image. Compared to Figure 3d, Figure 3e has almost no cross-structure. It shows that the missing of image discontinuities across the border of the image eliminates the effect of image border. The magnitude of Fourier Transform of smooth image (shown in Figure 3c) is shown in Figure 3f, which has almost only cross-structure information. Those figures illustrate the periodic image can eliminate the effect of image border and retain the information of original image to the greatest extent.

### 3.3. The Framework of Image Registration Based on Phase Correlation

**Figure 4:**
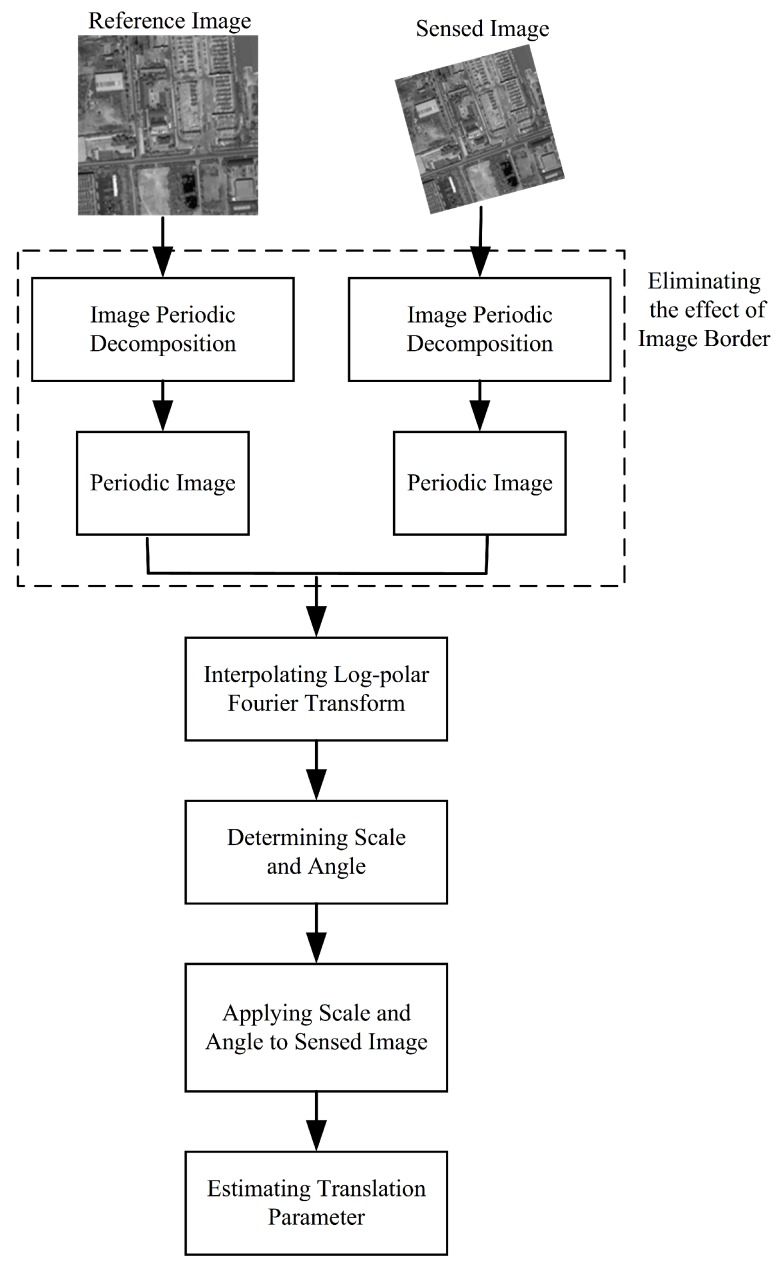
Overall workflow of the image registration based on phase correlation.

Here, we outlined the processing flow of phase correlation based image registration, including scalar, rotation angle and displacement estimation. The flowchart is shown in Figure 4. In addition, the description of each step is detailed as follows:

(1) Eliminating the effect of image border: decompose the reference image *R* and sensed image *S*, and acquire the corresponding periodic image $R^{\prime }$ and $S^{\prime }$. Its calculation process is detailed in the Section 3.2;
(2) Determine the scalar *s* and rotation angle θ: calculate the log-polar Fourier Transform of $R^{\prime }$ and $S^{\prime }$ by interpolating multi-layer fractional Fourier Transform [37]. Then, calculate their magnitude spectrum, respectively, and finally obtain the rotation angle θ and scalar *s* by determining the phase difference. For the multi-layer fractional Fourier Transform algorithm, the number of layers is 4, and the corresponding scalars are determined by the MATLAB function histcounts. For the histcounts, its input is the radius series values of points in a single radial line. For the construction of a log-polar grid, the number of radial lines is 128 and the number of points in each radial line is 128, and the logarithmic base is 1.044 and the minimum radius is set to 0.015. The interpolation approach for calculating the Log-polar Fourier Transform is the bicubic method. In the process of determing the scale and rotation, the involved displacement estimation is implemented by directly calculating the linear phase difference in the frequency domain and fitting the straight line by the usage of the least square method;
(3) Recover the sensed image $S^{\prime }$: according to the estimated rotation θ and scalar *s*, correct the angle and scale deformation, and acquire the corrected sensed image, which has only displacement with the reference image;
(4) Again, apply the phase correlation approach to obtain the translation [30]. The displacement estimation is implemented by directly calculating the linear phase difference in the frequency domain and fitting the straight line by the usage of the least square method.

## 4. Experiment and Analysis

To thoroughly analyze the role of different window functions and the proposed method (referenced as decomposition method), three subsets of experiments were carried out. The first one is about the comparison of success rate of image registration for different methods in the case of small patches’ registration. The second one and third one is about the comparison of registration accuracy for different methods in the case of displacement estimation as well as angle and scale estimation, respectively. In all of the following experiments, the parameter settings are the same. The roll-off factor, β, of the raised-cosine window is set to 0.25. The stretch parameter, *k*, of the flat-top window is set to 2.7.

### 4.1. The Comparison of Image Registration Success Rate for Different Methods

**Figure 5:**
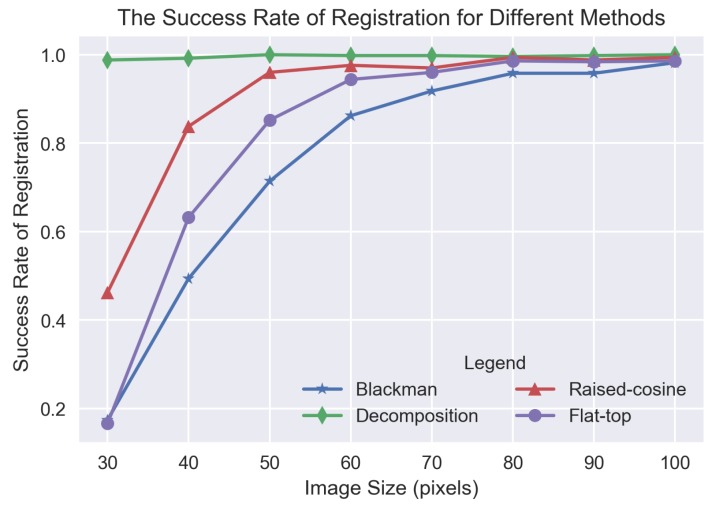
The success rate of registration for different methods.

Various sizes of remote sensing images were applied to evaluate the success rate of image registration for different methods of eliminating the effect of image border. There are eight groups of remote sensing images, whose sizes vary from 30×30 to 100×100 pixels in a step of 10. For each group, 500 images are included. The content they covered are diverse, including man-made buildings, mountain, residential area and so on. For each image, a corresponding translated version is generated by randomly translating a displacement, ranging from 1/3 to 2/3 the size of the image. For each generated image pair, we utilized Blackman, raised-window, flat-top and the decomposition method to eliminate the effect of image border, respectively; then, the displacement is acquired by the phase correlation based method. Considering the characteristic of high estimation accuracy of the phase correlation based algorithm, the criteria of registration success is that the absolute error between the true displacement and the estimated displacement in both *x*- and *y*-directions is less than one pixel. The experiment results are shown in Figure 5.

Apparently, regardless of the image size, the success rate of the decomposition method is highest and almost close to 1. For the other three methods, Blackman, raised-cosine and flat-top, the success rate is relatively low, especially when the image size is less than 50×50 pixels. Among the three window functions, the registration success rate curve of Blackman is below those of the other two window functions, and the curve of raised-cosine is above those of the other two window functions. The difference between the success rate of different methods is mainly due to the fact that the information near the image border is subjected to varying degrees of degradation. For the decomposition method, very little information is degraded, which results in its high success rate in the case of large displacement of small image registration. For Blackman window function, it degrades a large amount of information near the image border, which reduces its success rate heavily. Especially when large displacement exists between small size images, the blurring of a large amount of information near the image border leads to little common information left between images. As the image size grows gradually, the phenomenon will be reduced, and the success rate will increase a lot.

### 4.2. The Comparison of Displacement Estimate Accuracy for Different Methods

To compare the performance of different methods in the case of displacement estimation, two hundred remote sensing images with the size of 256×256 pixels were applied. They cover a wide variety of content, including farmland, road, river, buildings and so on. To generate an image pair, each image was translated randomly by a displacement ranging from 1 pixel to 1/4 size of image. Blackman, raised-cosine, flat-top and decomposition method were utilized to eliminate the effect of image border, respectively. The translation was estimated by using the phase correlation based method. The mean absolute error $e_{x}$ and $e_{y}$ in the *x*- and *y*-directions, and RMSE in the *x*- and *y*-directions are reported in Table 1.

From Table 1, in terms of the mean absolute error of displacement in the *x*- and *y*-directions, the proposed method, the decomposition method obtained minimal error, and the other three methods, Blackman, raised-cosine and flat-top method, are also hard to rank. In terms of RMSE of absolute error, the proposed method obtained the minimal RMSE in the *y*-direction and the second lowest RMSE in the *x*-direction. In summary, we can roughly conclude that the decomposition method has better performance than the other three methods in the case of only displacement estimation. The reason why the three window functions are difficult to rank is that the amount of degraded information affecting the estimation accuracy is also related to the displacement between image pairs. Some detailed discussion is given in Section 4.4.

### 4.3. The Comparison of Scale and Angle Estimation Accuracy for Different Methods

To compare the performance of different methods for eliminating the effect of image border in the case of scale and rotation transformation, a small subset of aerial images, from the USC-SIPI image database, are used in the test. There are thirty-seven images, whose sizes vary from 256×256 to 1024×1024, and contents include man-made buildings, ports, mountain and so on. We shifted, rotated and scaled each image randomly to acquire their corresponding transformed images. The range of displacement is from zero to one-quarter of the image size, and the scale factor varied from 0.1 to 0.9, and rotation angle is from 30${}^{\circ }$ to 90${}^{\circ }$. Here, three window function Blackman window, flat-top window and raised-cosine window weighting function and decomposition method were applied to eliminate the effect of image border. To quantitatively evaluate the performance of our proposed algorithm, two kinds of measurements are used. One is the success rate of registration in our test images subjected to certain transformation. The criteria of registration success is that the absolute error of scalar estimation is less than 0.01 and the absolute error of angle estimation is less than $2^{\circ }$. The other is the registration accuracy measurements, including the mean error and the root mean squared error (RMSE) of scalar and angle estimation in the case of registration success. To compare fairly, angle and scale factors are estimated by the same approach: multi-layer fractional Fourier Transform [37]. The results of each method are summarized in Table 2.

From Table 2, we can see that, for the value of the indicators of the proposed algorithm, the decomposition method is best, which showed the superiority of eliminating the effect of image border. As for other algorithms, it is hard to tell the superiority of them directly according to these measurements. The raised-cosine window acquired higher success rate than Blackman, but its mean error of scalar and angle is higher than the one of Blackman. Theoretically, a raised-cosine window should have better performance on eliminating the effect of image border while preserving more information, but its performance depends on the roll-off factor β parameter tuning. In addition, it is very challenging to find an optimal β for all the images in a subset; in particular, there are scale differences and angle rotation. The same problem also exists in the flat-top algorithm. Thus, our proposed algorithm is easiest to use and has the highest performance.

### 4.4. Further Analysis of Eliminating the Effect of Image Border Methods

To learn more deeply and thoroughly about the role of different window functions for eliminating the effect of image border, an experiment about images with different sizes of 64×64, 128×128, 512×512 and 1024×1024 pixels were applied to evaluate the displacement estimation accuracy for different methods of eliminating the effect of image border. The number of images for each image set with the same size is 200. For each image, randomly translating it ranging from 1 to 1/4 size of image to generate a image pair. Then, for each image pair, Blackman, flat-top, raised-cosine and the decomposition method were applied to eliminate the effect of image border, respectively. The displacement estimation accuracy is measured by the mean absolute error $e_{x}$ and $e_{y}$ in the *x*- and *y*-directions. The experiment results were reported in Table 3. For the images with 256×256 pixels, its result is directly referred from Section 4.2.

For images with smaller size of 64×64, 128×128 and 256×256 pixels, we can see that the decomposition method acquired the minimum error of $e_{x}$ and $e_{y}$, especially for the image size of 64×64 pixels. While for images with larger size, 512×512 or 1024×1024 pixels, the estimated error of the decomposition method is larger than the one of window functions. This shows that the decomposition method no longer has an advantage than weighted function methods in the case of image with larger size.

**Figure 6:**
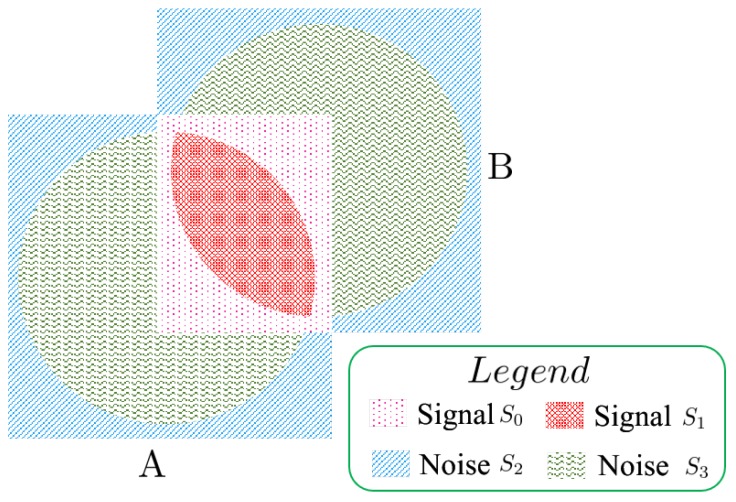
**A**,**B** represent two images, respectively. There exist displacements between them. Their corresponding weighted filtering window is denoted by a circle and centered at the image. Signal $S_{0}$ denotes the information in the overlap and suffered from the degradation due to the filtering of weighted window function. Signal $S_{1}$ denotes the information in the overlap and is free from the degradation due to the filtering of weighted window function. The difference between signal $S_{0}$ and $S_{1}$ is that one is degraded by the weighted window function and one is not. Noise $S_{2}$ denotes the information that is not in the overlap and suffered from the degradation due to the filtering of weighted window function. Noise $S_{3}$ denotes the information that is not in the overlap and is free from the degradation due to the filtering of weighted window function. The difference between noise $S_{2}$ and noise $S_{3}$ is that one is degraded by the weighted window function and one is not.

To explain the problem more clearly, we introduced a new concept of SSNR (Similar-to-Signal-Noise Ratio) for a pair of images, similar to signal-to-noise (SNR) in the field of signal processing. To explain the ANSR more clearly, a corresponding schematic diagram is shown in Figure 6. Here, we assume that there are two images A and B, and a displacement relationship between them. A and B represent two images, respectively. Their corresponding weighted filtered window denoted by a circle is centered at the center of each image. It is worth noting that, for each image, their content not in the scope of the circle will suffer from varying degrees of degradation due to the filtering of weighted window function. To simplify, we assume that the content in the common area of the two images is exactly the same, namely that there are no content changes in the overlapping region.

We divide the information into four categories and render each category by a different pattern. They are shown in Figure 6. For signal $S_{0}$, it denotes the information in the overlap and suffered from the degradation due to the filtering of weighted window function. For signal $S_{1}$, it denotes the information that is in the overlap and is free from the degradation due to the filtering of weighted window function. The difference between signal $S_{0}$ and $S_{1}$ is that one is degraded by the weighted window function and one is not. For noise $S_{2}$, it denotes the information that is not in the overlap and suffered from the degradation due to the filtering of weighted window function. For noise $S_{3}$, it denotes the information that is not in the overlap and is free from the degradation due to the filtering of weighted window function. The difference between signal $S_{2}$ and $S_{3}$ is that one is degraded by the weighted window function and one is not. The SSNR is expressed as $SSNR=\frac{S_{0}+S_{1}}{S_{2}+S_{3}}$. All of the weighted window function, image size and the displacements between images affect the $S_{0}$, $S_{1}$, $S_{2}$ and $S_{3}$.

Thus, the increase of SSNR is beneficial to the phase correlation-based image registration. When the SSNR becomes lower, it will reduce the estimation accuracy or even lead to the estimation failure of the phase correlation-based method. For large images with small displacements, after the operation of blurring by window function, the content retained in the central of images also has larger overlap. Thus, in this case, blurring these images may increase the SSNR because, although it degrades some information that has a corresponding piece in its transformed image, it also reduces the amount of the non-corresponding content of the image. Thus, the window function can not only eliminate the effect of image border, but also increase the SSNR, especially for the large images with small displacements. This is different from the existing understanding in the past research that the window function is only a tool of eliminating the effect of image border and produces side effects of reducing the amount of common information at the same time. The new understanding is helpful for researchers to select the best approaches of eliminating the effect of image border in different cases.

For the flat-top and raised-cosine method, their acquired estimation error for images with sizes of 64×64 are significantly larger than the ones of Blackman and decomposition. This is due to the constant parameter setting of these two methods for images with varied sizes. This illustrates that the related parameter setting of the flat-top and raised-cosine method is sensitive to image size. For the different parameter settings of flat-top and raised-cosine, they control the amount of information that is blurred, which affected the SSNR of image pairs finally. Thus, in the practical application, the performance is sensitive to the fine-tuning of parameters of flat-top and raised-cosine, which reduced the ease of application of these two methods.

Through the above analysis, we get a new understanding that the degradation of content near the image border affects the SSNR of a pair of images. All of these factors, the translation, the image size and the amount of information degraded by the window function, affect the ANSR of a pair of images. For small size image with larger displacement, as Section 4.1, the more the amount of information degraded, and the lower the SSNR of a pair of images, so the decomposition can obtain the highest success rate of registration. For image pairs with scale and rotation deformation, as Section 4.3, a relationship that has been established is that degrading the information by the window function can lower the ANSR. However, regarding how the amount of degraded information affects the ANSR, it is hard to determine directly and is related to the amount of scale and rotation. Thus, the performance of three window functions is hard to rank. For large images with small displacements, the larger the amount of information blurred by window function, the higher the ANSR. Thus, the Blackman window function gets the best performance and the decomposition method has the worst performance. As for other cases, it is hard to directly determine the relationship between the amount of blurred information and the value of SSNR.

## 5. Discussion

To illustrate the advantages of the proposed method, the decomposition method for image registration based on phase correlation, three groups of experiments were carried out and the other three methods, raised-cosine window, Blackman window and flat-top window, were introduced for comparison. For the first group experiment, we evaluated the displacement estimation success rate of different methods for different sizes of images. The decomposition method always achieved the stable and high success rate than other methods for different sizes of image, especially for images with sizes less than 50×50 pixels. For the second experiment, we compared the different mean absolute errors in the *x*- and *y*-directions $e_{x}$, $e_{y}$ and RMSE in the *x*- and *y*-directions, $x_{RMSE}$ and $y_{RMSE}$. The experiment results show that the decomposition method can always achieve the minimum error except for the measurement of $x_{rmse}$, which is the second minimum, compared to other methods. The third experiment is about the estimation of scale and angle. The experimental results show that the decomposition method can acquire a higher success rate, a smaller absolute error and RMSE for the estimation of scale and angle. In addition, although all the test images in the experiment are square, the decomposition method is independent of the image aspect ratio because the decomposition method is carried out by the rigorous mathematical formula and all the involved mathematical operations are also independent of image aspect ratio.

Additionally, another group experiment of displacement estimation of images with five different sizes were carried out to give more insights on eliminating the effect of image border. An interesting experiment result was reported in which the decomposition method has a larger estimation error than weighted window filter methods in the case of images with a size of 1024×1024. Through the in-depth analysis, a new concept of SSNR, the ratio of the corresponding image content information to the non-corresponding image content information was introduced to interpret the results and a novel understanding of the role of weighted window filter function was gained. The window function can not only eliminate the effect of image border, but also affect the SSNR of image pair at the same time. The increment of SSNR is beneficial to image registration based on phase correlation. However, the value of SSNR is related to the image size, displacement and the amount and distribution of image content changes. Thus, in the practical application, it is difficult to select a general window function with fixed parameters to acquire the best performance in the different cases, while the decomposition method is easy to use and no parameters are required for tuning.

As for the efficiency of the proposed method, the image periodic decomposition method, its time complexity is mainly dependent on the calculation of smooth image component. Because the original image is equal to the sum of the periodic image and smooth image component, we can acquire the smooth component firstly and then acquire the periodic component by subtracting a smooth component from the original image—while, for solving a smooth component, its time complexity is O(M∗Nlog(M∗N)), which is the same as the time complexity of the phase correlation based image registration method. Here, *M* and *N* denote the width and height of image, respectively. Thus, the complexity of the proposed method is also O(M∗Nlog(M∗N)) [35].

## 6. Conclusions

In this paper, we proposed a novel algorithm of eliminating the image border, namely image periodic decomposition technology. Different from traditional methods of alleviating the effect of image border, it decomposed the original image into periodic image and smooth image by reformulating the periodic image as a unique solution for a specified problem and using the Fourier Transform based Poisson solver to acquire the periodic image. The smooth image is the difference between the original image and periodic image. The periodic image had no effect of border when calculating Fourier Transform and kept the original image information to the maximum extent. This can improve the image registration accuracy and success rate in small size images with large translation, or in the case of scale and rotation. Moreover, it can always get the ideal periodic image no matter what size and content an image has, and there are no hyper-parameters to tune. Additionally, by the introduction and comparison of the decomposition method, we further understand the role of window function. The amount of information degraded by the window function, together with the deformation between image pairs, affect the SSNR of image pairs, and the high SSNR is beneficial to the phase correlation based image registration. In future work, we will explore the relationship between the amount of degraded information and SSNR in the various cases of varying sizes of image pairs with different translation, rotation and scale transformation.

## Figures and Tables

**Table 1:** The comparison of performance for different methods of eliminating the effect of image border in the case of estimating the displacement.

Algorithm	$e_{x}$	$e_{y}$	$X_{\mathrm{RMSE}}$	$Y_{\mathrm{RMSE}}$
Decomposition	0.263	0.248	0.0195	0.0197
Blackman	0.268	0.249	0.0190	0.020
Flat-top	0.266	0.251	0.0195	0.021
Raised-cosine	0.265	0.254	0.0196	0.0216

**Table 2:** Comparison of the performance of each algorithm in estimating scalar and angles.

	Algorithm	Decomposition Method	Raised-Cosine Window	Blackman Window	Flat-Top Window
Measurements					
Success rate total images (37)		33/37	22/37	20/37	17/37
Mean scalar error (unit: $10^{-3}$)		2.40	3.19	2.64	3.64
Mean angle error (unit: $10^{-1}$)		1.47	8.86	5.41	9.55
RMSE of scalar (unit: $10^{-5}$)		0.66	1.69	1.34	2.12
RMSE of angle (unit: angle(°))		0.04	1.05	0.57	1.18

Notes: In the calculation of success rate, the total number of test images is 37 pairs, and the success rate is equal to the ratio of the number of matched image pairs to the number of total image pairs.

**Table 3:** The comparison of displacement accuracy for different methods of eliminating the effect of image border in the case of images with different sizes. $e_{x}$ and $e_{y}$ denote the mean absolute error in the *x*- and *y*-directions, respectively.

	Algorithm	Decomposition		Blackman		Flat-top		Raised-cosine	
Image Size		$e_{x}$	$e_{y}$	$e_{x}$	$e_{y}$	$e_{x}$	$e_{y}$	$e_{x}$	$e_{y}$
64×64		0.244	0.240	0.266	0.250	0.294	0.282	0.291	0.292
128×128		0.248	0.251	0.252	0.253	0.256	0.263	0.254	0.254
256×256		0.263	0.248	0.268	0.249	0.266	0.251	0.265	0.254
512×512		0.253	0.251	0.252	0.251	0.253	0.250	0.251	0.252
1024×1024		0.246	0.270	0.243	0.241	0.243	0.250	0.242	0.245
